# Transplanted olfactory ensheathing cells restore retinal function in a rat model of light-induced retinal damage by inhibiting oxidative stress

**DOI:** 10.18632/oncotarget.21857

**Published:** 2017-10-16

**Authors:** Langyue Xue, Yuxiao Zeng, Qiyou Li, Yijian Li, Zhengya Li, Haiwei Xu, Zhengqin Yin

**Affiliations:** ^1^ Southwest Hospital/Southwest Eye Hospital, Third Military Medical University, Chongqing 400038, China; ^2^ Key Lab of Visual Damage and Regeneration & Restoration of Chongqing, Chongqing 400038, China

**Keywords:** light damage, photoreceptor, olfactory ensheathing cells, reactive oxygen species

## Abstract

There is still not an effective treatment for continuous retinal light exposure and subsequent photoreceptor degeneration. Olfactory ensheathing cell (OEC) transplantation has been shown to be neuroprotective in spinal cord, and optic nerve injury and retinitis pigmentosa. However, whether OECs protect rat photoreceptors against light-induced damage and how this may work is unclear. Thus, to elucidate this mechanism, purified rat OECs were grafted into the subretinal space of a Long-Evans rat model with light-induced photoreceptor damage. Light exposure decreased a- and b- wave amplitudes and outer nuclear layer (ONL) thickness, whereas the ONL of rats exposed to light for 24 h after having received OEC transplants in their subretinal space was thicker than the PBS control and untreated groups. A- and b- wave amplitudes from electroretinogram of OEC-transplanted rats were maintained until 8 weeks post OEC transplantation. Also, transplanted OECs inhibited formation of reactive oxygen species in retinas exposed to light. *In vitro* experiments showed that OECs had more total antioxidant capacity in a co-cultured 661W photoreceptor cell line, and cells were protected from damage induced by hydrogen-peroxide. Thus, transplanted OECs preserved retinal structure and function in a rat model of light-induced degeneration by suppressing retinal oxidative stress reactions.

## INTRODUCTION

Continuous retinal light exposure may irreversibly damage photoreceptor cells [1], and this is a common eye insult [2, 3]. Although the pathogenesis of light-induced retinal damage is unclear, several pathological changes, including elevated calcium ions [4], apoptosis, free radical production and lipid peroxidation-have been reported to be involved in irreversible retinal damage after exposure to intense light [5]. Currently, the main therapeutic methods for light-induced retinal damage involve treatment with calcium-channel-blockers [6], glucocorticosteroids [7], neurotrophic factors [8, 9], antioxidant and anti-lipid peroxidation treatment [10, 11], but their effects are not satisfactory.

Olfactory ensheathing cells (OECs) are a special group of glial cells that reside in the olfactory system and support neurogenesis throughout a person's life time [12]. These cells share some characteristics with Schwann cells and astrocytes. Like astrocytes, OECs express glial fibrillary acidic proteins (GFAP) in their cytoskeleton, but they share similar morphological and molecular markers with Schwann cells [13–16]. OECs may secrete various neurotrophic factors, including nerve growth factors (NGFs), brain derived neurotrophic factors (BDNFs), glial cell line-derived neurotrophic factors (GDNFs), ciliary neurotrophic factors (CNTFs) and vascular endothelial growth factors (VEGFs). Also, OECs express proteins such as S-100, nerve growth factor receptor p75 (NGFR p75), neural cell adhesion molecule (N-CAM) and laminin, all of which contribute to the development of neurons [15, 17]. Recent studies indicate that grafted OECs rearranged glial scars in CNS and provided tunnels for the regeneration of axons after adult mammals spinal cord injury [18, 19]. Transplantation of OECs into CNS lesion induced axonal regeneration and neural functional recovery [14, 20–27]. OECs can enter fibroblast zones to promote neurite outgrowth in growth-inhibitory areas in the multicellular scar-like culture model [28]. More effective axon regeneration and functional recovery of the CNS are usually achieved via transplantation of genetically engineered OECs [29]. It has also reported that OECs improve local microcirculation of the damaged spinal cord and promote angiogenesis, thus enhancing remyelination of demyelinated neuronal axons [30, 31]. According to previous work, OECs transplantation is clinically safe [32, 33] and offers promising therapeutic outcomes for patients [34].

In the visual system, grafted OECs promote regeneration of retinal axons of ganglion cells in adult rats with optic nerve crush [35]. OECs are incorporated into the optic nerve head after transplantation into the retina of the retrobulbar optic nerve after axotomy in rat models [36]. Leaver's group [37] reported that OECs could support the regrowth of retinal ganglion cell neurites *in vitro*. Our previous results indicated that grafted OECs in the subretinal space of Royal College of Surgeons (RCS) rats migrated through all layers of the retina and suppressed gliosis formation by the over-activation of Müller cells [38]. We found also that purified OECs inhibited activation of Müller cells by suppressing the Notch pathway, thus protecting photoreceptors in RCS rats [39].

Thus, grafted OECs could protect recoverin-positive photoreceptor cells and delay retinal degeneration [40]. However, whether OECs also protect against light-induced retinal damage and its mechanisms is unknown. Therefore, we used a rat model with light-induced retinal damage to explore the effect of grafted OECs on retinal light damage. Then, we used 661W photoreceptor cells to investigate the ability of OECs to protect against oxidative stress-induced damage and attempted to elucidate the underlying mechanism.

## RESULTS

### Effect of light exposure on retinal morphology and function in Long-Evans rats

**Figure 1:**
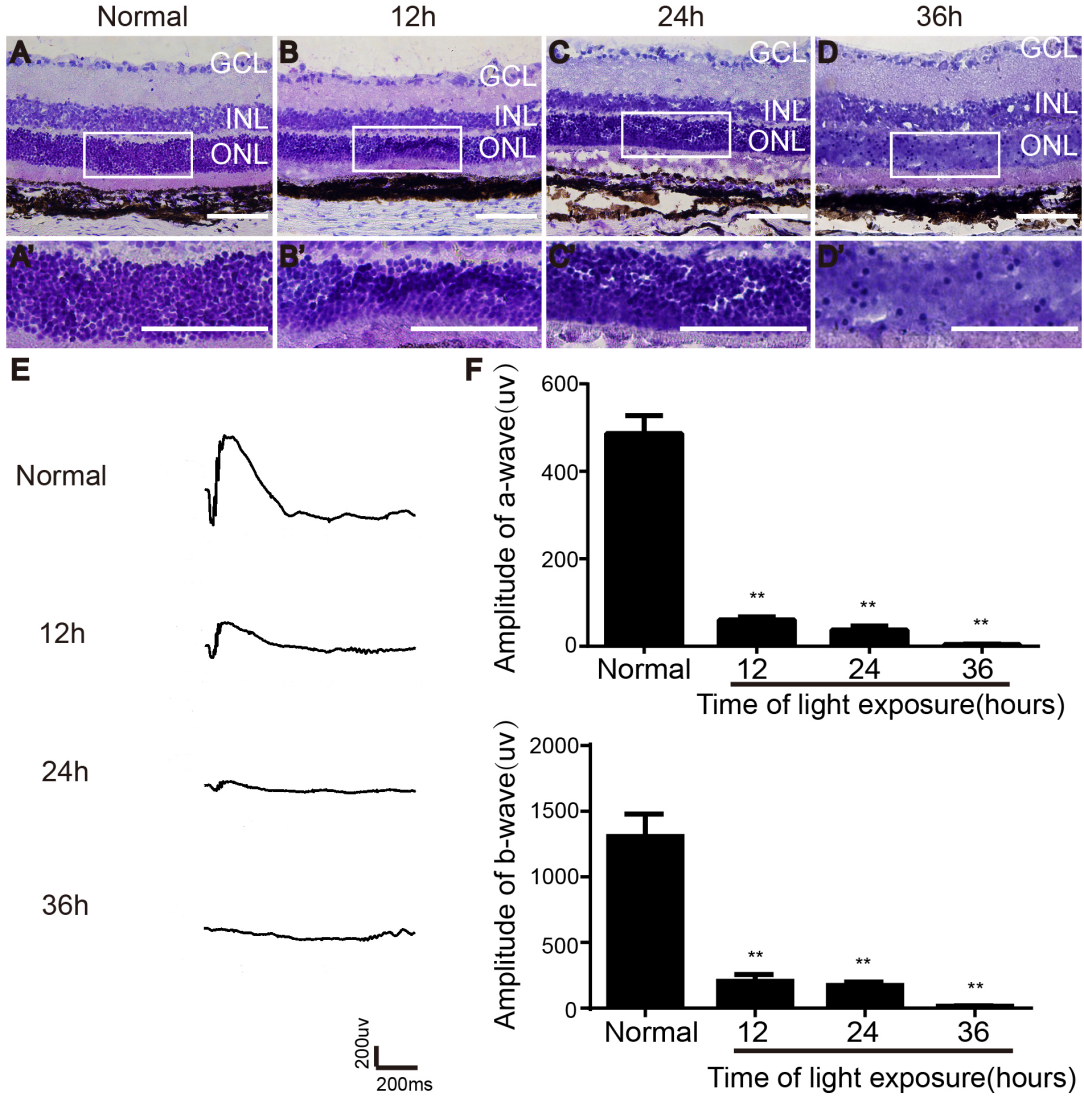
Effect of light exposure on retinal morphology and function in LE rats **(A-D)** Morphological changes of retina after different times of light exposure. **(E)** ERG traces recorded after different time of light exposure. **(F)** Amplitudes of a- and b-wave.GCL, ganglion cell layer; INL, inner nuclear layer; ONL, outer nuclear layer. Scale bars, 100 μm. n=5, ^**^, *p* < 0.01.

To evaluate the effect of light exposure on the retina, electroretinogram (ERG) recording and hematoxylin and eosin (H&E) staining were performed one day after light exposure. H&E staining showed that the organized structure of retina in unexposed rats was intact (Figure 1A). After 12 h of light exposure, cells in the ONL of the retina were irregularly arranged, and few had hyperchromatic nuclei (Figure 1B). Twenty-four hours of light exposure caused more pyknotic nuclei, intercellular space dilatation and disordered-arrangement of ONL cells (Figure 1C). After 36 h of light exposure, the structure of ONL was more disordered, and most of the nuclei in the ONL become karyolytic and detached (Figure 1D). Simultaneously, the scotopic ERG amplitude decreased in the light-damaged groups. Compared with controls, the amplitude decreased by more than 80% in groups exposed to light for both 12 and 24 h. In particular, in the group exposed to light for 36 h, the amplitude was significantly diminished (Figure 1E and 1F). Thus, rats exposed to light for 24 h were selected for subsequent cell transplantation experiments.

### Isolation of OECs and protection of light-exposed retinas

**Figure 2:**
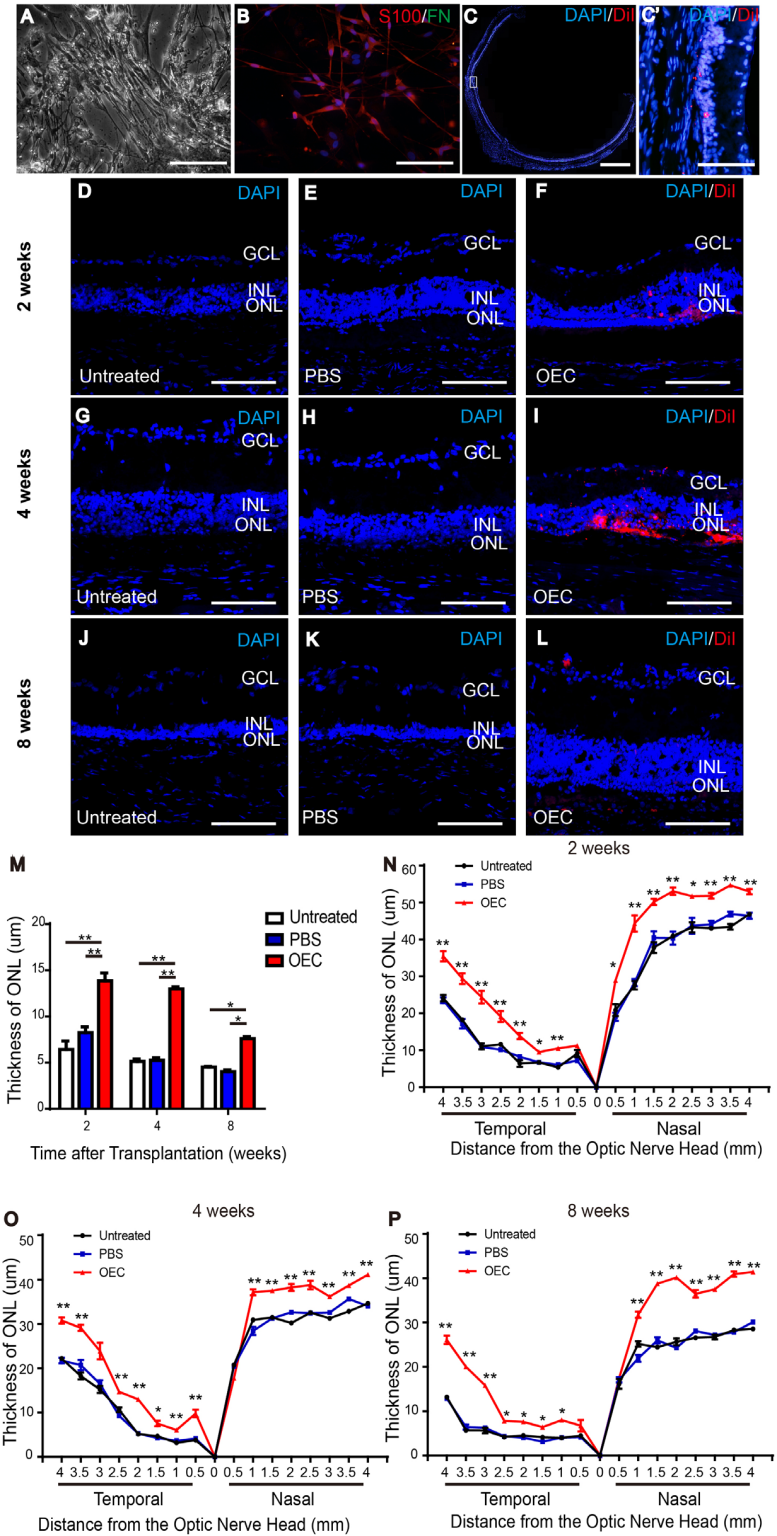
Characterization of OECs and protection of the ONL of light-damaged retinas of LE rats **(A)** Fusiform cells and flattened cells. **(B)** Representative images of OECs stained with S100 (red), fibronectin (green) and DAPI (blue). **(C)** Spread of the transplanted cells (red) in the subretinal space around the injection site. **(D, G, J)** Untreated rat at 2 weeks (D), 4 weeks (G) and 8 weeks (J). **(E, H, K)** PBS injection at 2 weeks (E), 4 weeks (H) and 8 weeks (K). **(F, K, L)** Retina with transplanted OECs at 2 weeks (F), 4 weeks (K) and 8 weeks (L). **(M)** Thickness of ONL in the temporal region 2 mm to the optic nerve head. **(N-P)** ONL thickness in the temporal and nasal regions of retina. Counterstained DAPI, blue; transplanted cells CM-DiI, red. Scale bars, 100 μm (A, B, C'-L), 1000 um (C). n=3, ^*^, *p* < 0.05, ^**^, *p* < 0.01.

After two weeks, primary cultured OECs grew tightly packed in fusiform or spindle forms (Figure 2A). S100- positive OECs were characterized by primarily fusiform or spindle forms, while fibronectin (FN)-positive olfactory nerve fibroblasts (ONFs) had diverse forms (Figure 2B). After purification, approximately90% of cells were S100-positive OECs.

Eight weeks post-transplantation, CellTracker CM-DiI-labeled cells spread in the subretinal space (Figure 2C). The ONL of the temporal retina in the light-induced retinal damage group gradually thinned over time after light exposure (Figure 2D-2M). At 2, 4, and 8 weeks, the ONL thickness for every 0.5 mm intervals in the OEC group was significantly thicker than that in the PBS and untreated groups in the temporal retina (the injection region) and the nasal retina (Figure 2N-2P).

### Transplanted OECs and ERG recording of light exposure-induced retinal degeneration

**Figure 3:**
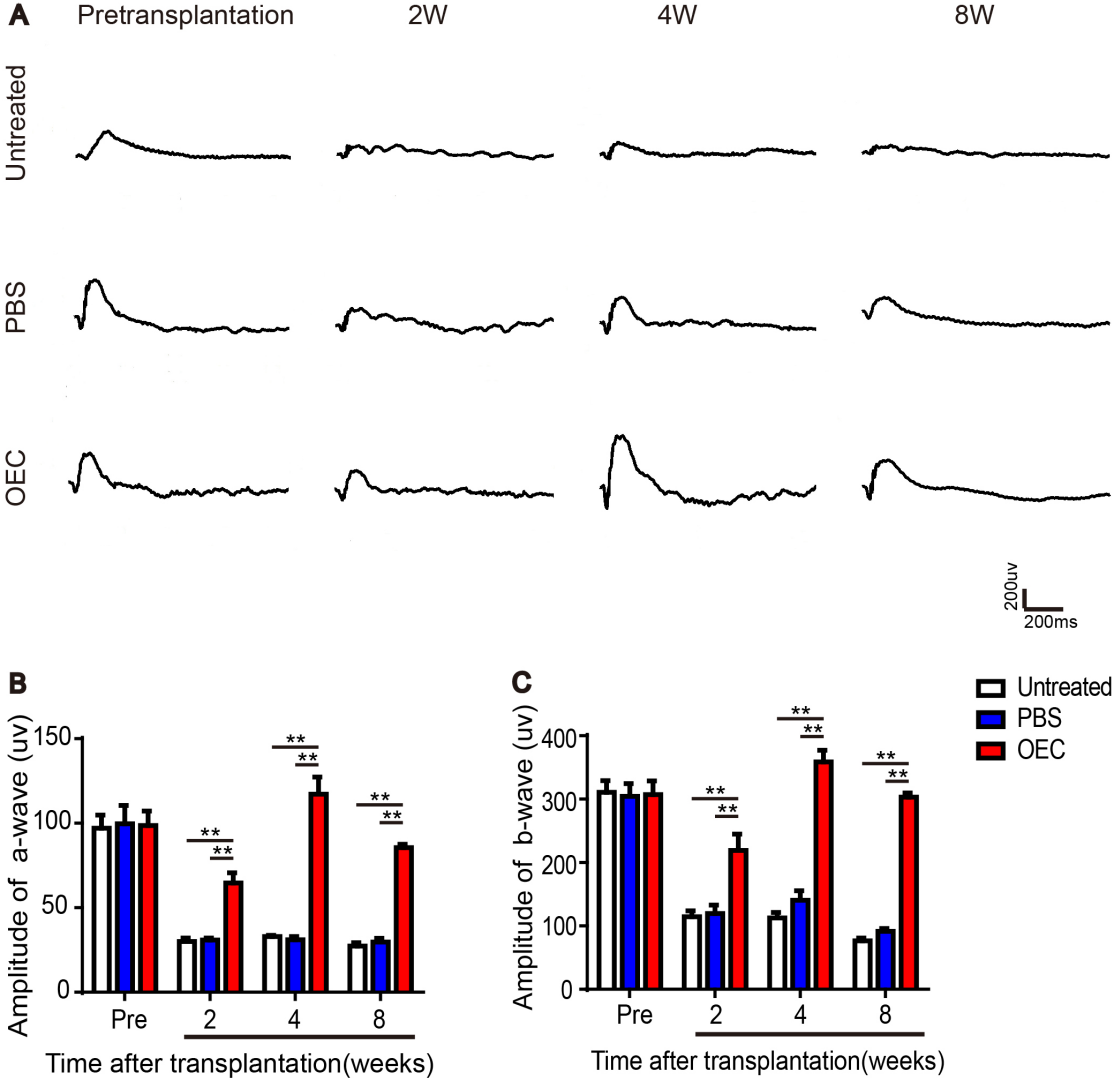
Transplanted OECs rescue ERG recordings of light exposure-induced retinal degeneration **(A)** ERG traces after transplantation of OECs. **(B)** Amplitudes of a-wave. **(C)** Amplitudes of b-wave. n=6, ^*^, *p* < 0.05, ^**^, *p* < 0.01.

After light damage, a- and b-wave amplitudes of the untreated group decreased over time (Figure 3A). At 2 weeks, all groups had attenuation, but the amplitude of the OEC group was significantly different compared to all other groups, (*p*< 0.01) (Figure 3B and 3C). Four weeks after treatment, the amplitude of the OECs and PBS groups mildly rebounded, but the OEC group recovered more significantly, with significant differences (*p*< 0.01) compared to PBS and untreated groups (Figure 3B and 3C). At 8 weeks, amplitudes decreased in each group. However, the OEC group was significantly different compared to the untreated and PBS groups (*p*< 0.01) (Figure 3B and 3C). These results suggested that OEC transplantation improved visual function of degenerative retinas. Thus transplanted OECs delayed light exposure-induced photoreceptor degeneration.

### Effect of grafted OECs on reactive oxygen species (ROS) in light exposure-induced damaged retinas

**Figure 4:**
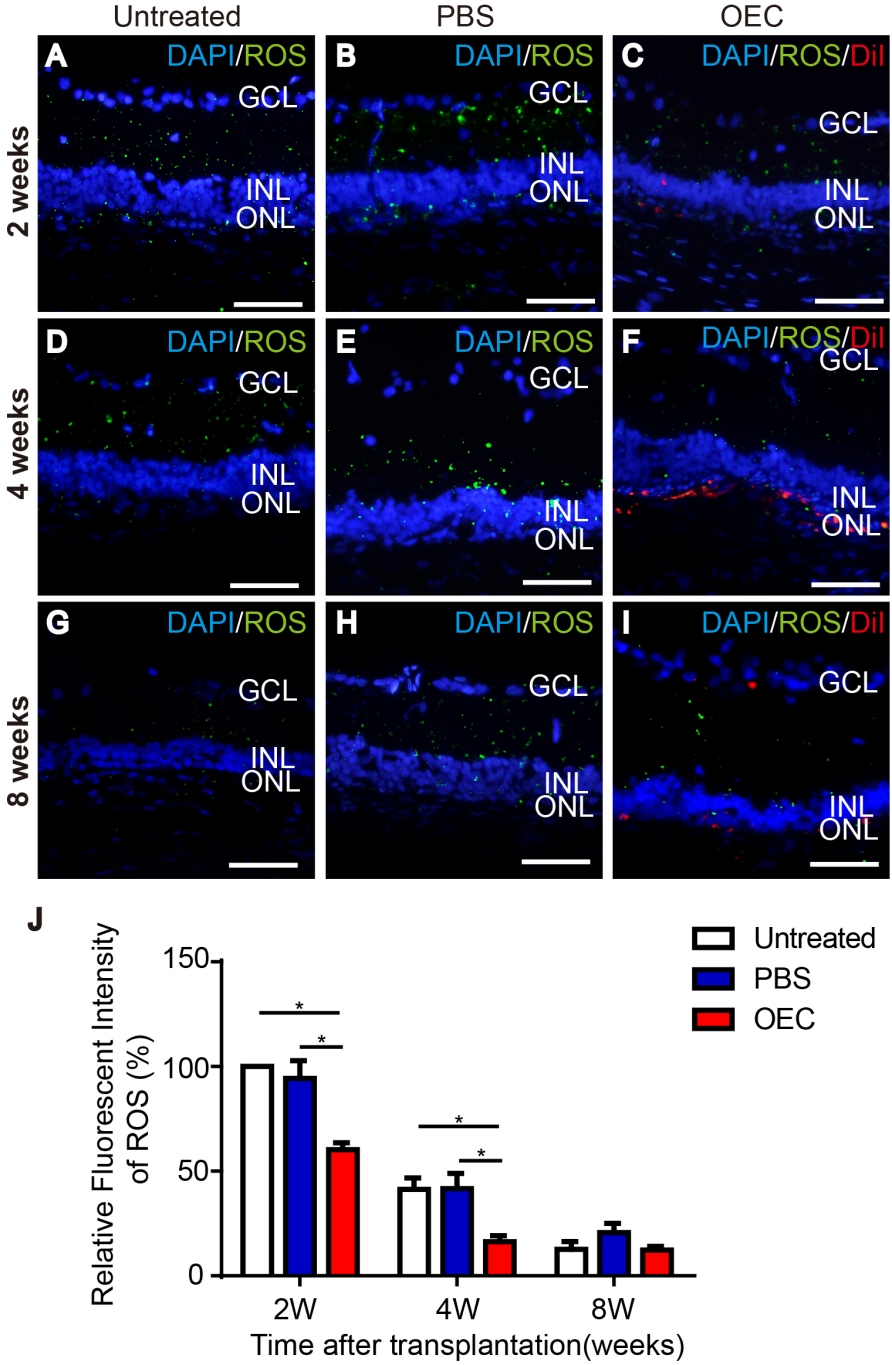
Effect of grafted OECs on the ROS level in light exposure-induced retina damage **(A, D, G)** Untreated rat at 2 weeks (A), 4 weeks (D) and 8 weeks (G). **(B, E, H)** PBS injection at 2 weeks (B), 4 weeks (E) and 8 weeks (H). **(C, F, I)** Retina with transplanted OECs at 2 weeks (C), 4 weeks (F) and 8 weeks (I). **(J)** Relative fluorescent intensity of ROS. Transplanted cells, red; ROS, green; Counterstained DAPI, blue. Scale bars, 50 μm. n=3, ^*^, p< 0.05.

ROS immunofluorescent staining showed that light damage produced peroxide in the retina (Figure 4A-4I). At 2 weeks, compared with PBS and untreated groups, peroxide labelling by 2′, 7′-dichlorofluorescein diacetate (DCFH-DA) in the retina of the OEC transplanted group had decreased (Figure 4A-4C and 4J) (*p*< 0.05). After 4 weeks, peroxide produced in the retina of all groups was decreased, but labeled peroxide in the OEC transplanted group was the lowest of all groups (Figure 4D-4F and 4J) (*p*< 0.05). After 8 weeks, the groups were not significantly different (Figure 4G-4I and 4J). Transplanted OECs thus could inhibit oxidative stress in the retina which was protective.

### Co-culture with purified OECs protects 661W from damage induced byH_2_O_2_

**Figure 5:**
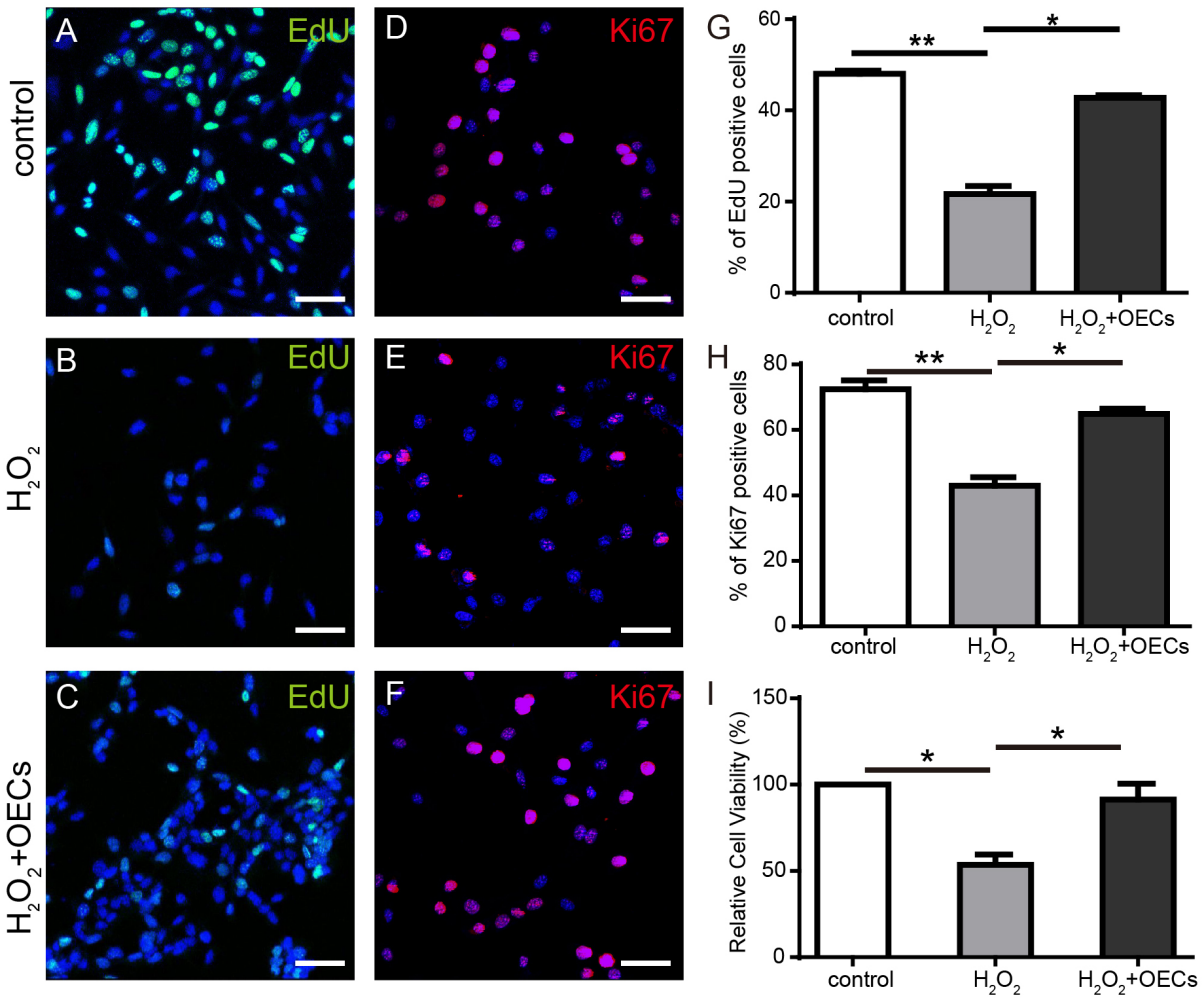
Co-cultured OECs protect 661W from proliferation impairment induced by H_2_O_2_ **(A-C** and **G)** EdU labeling analysis of 661W cells, n=3. **(D-F** and **H)** Ki67 staining of 661W cells, n=3. **(I)** CCK-8 assay analysis of 661W cells, n=5. Scale bars, 50 μm. ^*^, p< 0.05, ^**^, p<0.01.

5-ethynyl-20-deoxyuridine (EdU) and Ki67 staining showed that H_2_O_2_ exposure affected the proliferation rate of 661W cells. After exposure to 0.5mM H_2_O_2_ for 30 min, EdU and Ki67 positive cells significantly decreased, with then co-culturing of the OECs for 24 h mitigating the impacts of H_2_O_2_ (Figure 5A-5H). CCK-8 assay analysis indicated that the viability of 661W cells decreased approximately 50% after H_2_O_2_ exposure. However, co-culture with OECs for 24 h reduced damage to 661W cells induced by H_2_O_2_ (Figure 5I).

### Co-culture with OECs can mitigate apoptosis induced by H_2_O_2_

**Figure 6:**
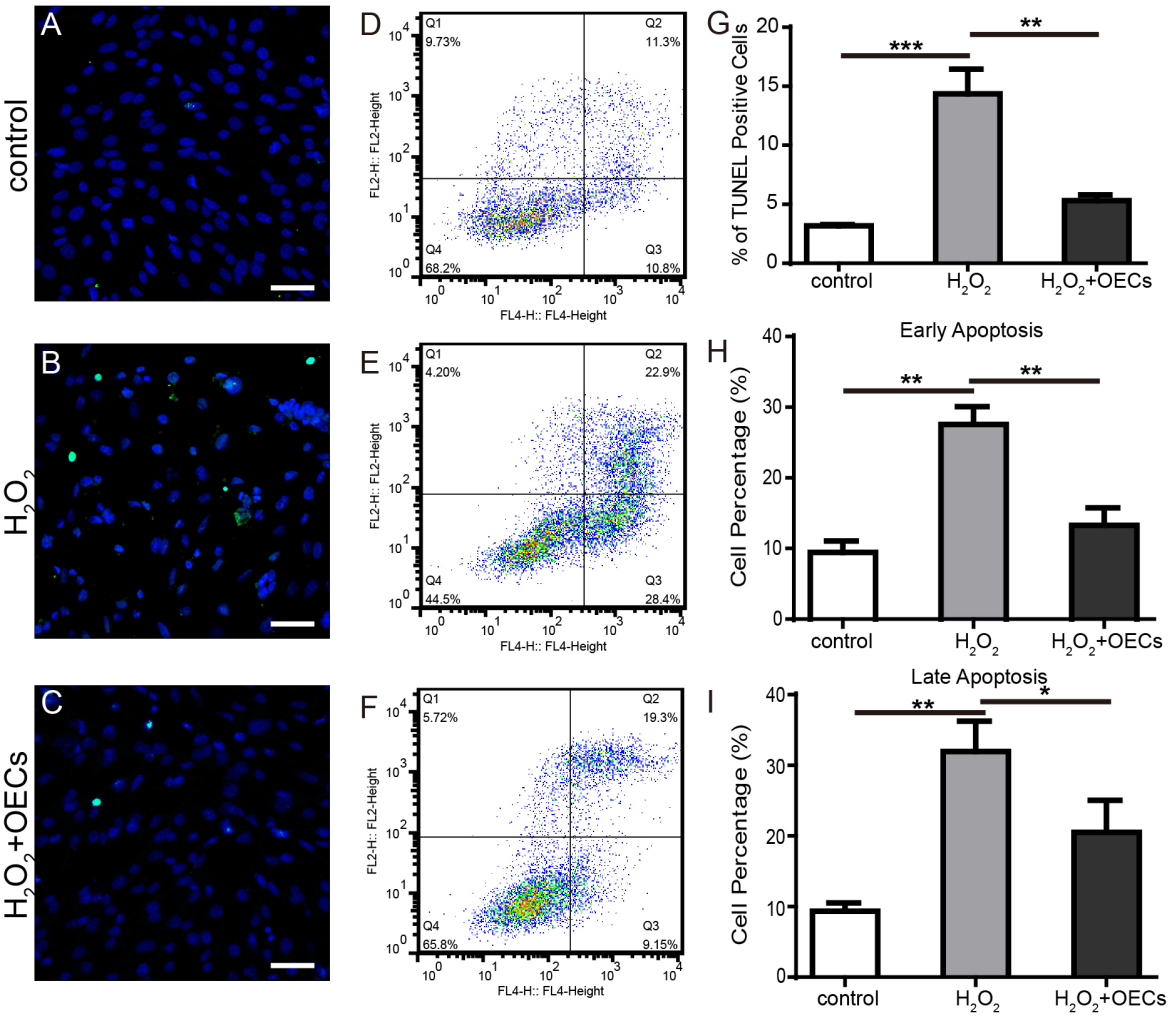
Co-culture with OECs mitigated apoptosis induced by H_2_O_2_ **(A-C)** Representative images of 661W cells stained with TUNEL (green) and DAPI (blue). **(D-F)** Flow cytometry analysis of apoptosis. **(G)** Statistics of TUNEL positive cells. **(H** and **I)** Statistics of flow cytometry analysis. Scale bars, 50 μm. n=3, ^*^, *p* < 0.05, ^**^, *p* <0.01, ^***^, *p* <0.001.

Exposure to H_2_O_2_ can increase TdT-Mediated dUTPNick End Labeling (TUNEL) positive cells, and co-culture with OECs diminish the proportion of them (Figure 6A-6C and 6G). Apoptosis of 661W cells was measured by flow cytometry and early and late apoptosis increased after exposure to H_2_O_2_, but this was reduced with co-culture with OECs(Figure 6D-6F and 6H-6I).

### OECs reduced intracellular ROS elevation after H_2_O_2_ exposure

**Figure 7:**
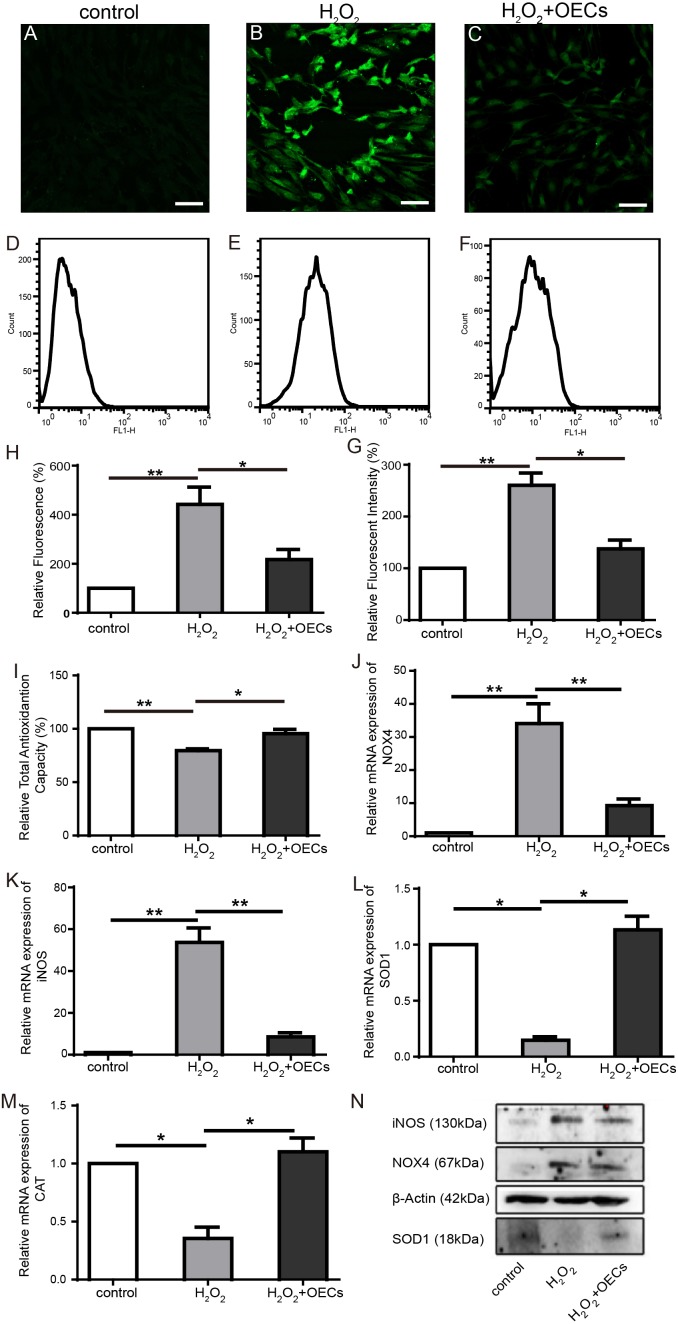
OECs alleviate elevation of intracellular ROS level induced by H_2_O_2_ exposure **(A-C** and **H)** Representative images of 661W cells stained with ROS (green) and statistics of relative fluorescent intensity of images, n=3. **(D-F** and **G)** Flow cytometry analysis of intracellular ROS production in 661W cells and statistics of relative fluorescent intensity, n=3. **(I)** Statistics of relative total antioxidantion capacity of 661W cells. **(J-M)** Relative mRNA expression of NOX4, iNOS, SOD1, CAT, n=5. **(N)** Western blot analysis. Scale bars, 50 μm. ^*^, p< 0.05, ^**^, p<0.01.

H_2_O_2_-induced oxidative stress response can induce cellular injury, so we assayed co-cultured 661W cells with DCFH-DA, and fluorescent intensity was quantified. Figure 7 showed that fluorescent intensity significantly increased in 661W cells after exposure to H_2_O_2_ for 30 min. Compared to the H_2_O_2_-treated group, co-culture with OECs reduced the accumulation of intracellular ROS. Co-culturing with OECs increased cellular antioxidant defense (Figure 7I).

Real-time PCR (Figure 7J-7M) showed that the expression of NOX4 and iNOS were significantly increased and the expression of SOD1 and CAT were decreased in the cells with H_2_O_2_ treated. However, after co-cultured with OECs, the expression of NOX4 and iNOS were markedly lower than in H_2_O_2_ group, and the expression of SOD1 and CAT were higher than in H_2_O_2_ group. Western blots showed that in the H_2_O_2_ group, the protein content of NOX4 and iNOS in H_2_O_2_ group were the highest and SOD1 content were lowest among all the samples (Figure 7N). Our results suggested that reduction of intracellular ROS by transplanted OECs may be an important protective mechanism for light damaged photoreceptors.

## DISCUSSION

In this study, we established a rat model for light-induced retinal degeneration according to published methods [41] and provided evidence (by immunofluorescent staining) that transplanting primary cultured OECs into the subretinal space might preserve both the retinal function and the ONL thickness in rats by mitigating retinal oxidative stress reactions with ERG. Our *in vitro* study confirmed that OECs could protect cells from H_2_O_2_-generated injury by alleviating the oxidative stress response.

A light stimulus signal triggers the visual transduction pathway by photoreceptors, but intense visible light can activate oxygen free radicals to damage the retina [2]. Retinal light-damage models have been used to study human retinal degenerative diseases caused by the environmental, age or genetic lesions, such as aged macular degeneration, in which retinal anatomical structural changes are similar to retinal cell remodelling during late stages of light damage in rodents [42–44]. Therefore, we used a light-induced retinal damage rat model. ERG can record animal retinal function, and this technique is used to observe the effect of experimental factors on retinal function. We used ERG to confirm the degree of light-induced retinal damage. Because light exposure usually causes regional decline in ONL thickness [45–47]. We made morphological observations; these approaches are consistent with the previous studies [47, 48].

Current studies of OECs and CNS repair suggest that OECs can induce a pathway for olfactory nerve fiber regrowth, migrate through glial scars, and produce phagocyte fragments [17, 18, 49]. OECs may express NGF, BDNF, GDNF, and CNTF as well as their receptors and secrete SCARB2 protein [50–53]. OECs can reduce astrocyte reactivity, promote axon regeneration and enhance functional recovery [20, 26, 54, 55].

Studies have shown that retinal photoreceptor outer segment membrane discs contain rhodopsin and long-chain polyunsaturated fatty acids, whereas the inner segments contain mitochondria, with highoxygen tension making them vulnerable to free radical attack [56]. Strong light may lead to retinal oxidative stress and cause peroxidation of the lipids of the photoreceptor outer segment membrane disc [57]. Absorbing visible light, rhodopsin produces a series of ROS, including superoxide anion radicals (O^2−^), hydroxyl radicals (HO^−^), and H_2_O_2_. These free radicals damage cell membrane lipids, proteins and nucleic acids and induce lipid peroxidation, producing toxic products that can induce apoptosis or necrosis and even dissolve the biofilm. The nicotinamide adenine dinucleotide phosphate (NADPH) oxidase (NOX) family contain enzymes that are known to generate ROS [58], and recent studies show that the NOX family contributes to generation of ROS in the retina [59, 60]. Nitric oxide (NO), a nitrogen radical, is known to be involved in the injury of light induced retina degeneration [61]. Nitric oxide synthase (NOS) family isozymes are known to mediate NO generation [62]. Under physiologic conditions, intracellular protective reductases, such as superoxide dismutase (SOD) and catalase (CAT), may clear ROS; however, when ROS abnormally increases, oxidative and antioxidant systems become imbalanced, leading to damage [5]. Recent studies show that OEC-conditioned medium protected astrocytes from damage by H_2_O_2_ and decreased the ROS and Ca^2+^ in astrocytes i*n vitro* [63]. OEC-conditioned medium may also promote antioxidant defense, leading to suppression of 6OHDA-induced oxidative damage by enhancing Akt survival signalling [64].

We have demonstrated that transplanting OECs into the subretinal space of rats with light-induced retinal damage reduced this damage and the loss of photoreceptors; it also preserved the ERG a-wave. ERG output might be influenced by injury potentials, but this is unclear [65]. We hypothesized that injury produced by the subretinal injection of PBS might activate retinal regeneration and contribute to rapidly improved ERG amplitudes. However, data showed that ERG amplitudes of the PBS group were not significantly higher than those of the untreated group at 2 and 4 weeks after transplantation. After transplanting OECs, ROS generation was significantly reduced. Grafts of OECs into the subretinal space decreased ROS in light-damaged retinas. The data for *in vitro* experiments showed that H_2_O_2_ damaged 661W cells by increasing NOX4 and iNOS and by the accumulation of intracellular ROS. After being co-cultured with OECs, NOX4 and iNOS expression decreased, SOD1 and CAT increased; and production of intracellular oxidative stress was decreased. Thus, oxidative damage to 661W cells was minimized. Improvement of total antioxidant capacity by co-culture with OECs suggested that OECs might reduce ROS by enhancing the antioxidant capacity of 661W cells.

These results suggest that oxidative stress might contribute to light-induced retinal damage and that transplanted OECs might scavenge free radicals, increase antioxidant capacity, and inhibit oxidation to protect photoreceptor cells, thus protecting them against light-induced retinal damage.

In summary, we offer an experimental basis for the use of OECs in light-damaged retinas and expanded the potential value of clinical applications of transplanted OECs in degenerative retinal diseases. Future work should include developing asource of OECs for clinical use and extend their role.

## MATERIALS AND METHODS

### Animals

Adult Long Evans rats (male and femail, ∼250g) were obtained from the Animal Research Center of the Third Military Medical University. All animal experimental procedures were conducted by adhering to the Association for Research in Vision and Ophthalmology Statement and were subjected to the provisions of the Southwest Hospital Animal Ethics Committee in Chongqing, China. During this study, the rats were housed in an air-conditioned room with dim cyclic light and *ad lib* food and water.

### Model of light-induced retinal damage

A rat model of light-induced retinal damage was adapted from published methods [41]. Then, after pupils were dilated with atropine sulphate ointment (Shanghai General Pharmaceutical Co., Ltd., China), the eyes of adult Long Evans rats (30 to 35 days old) were exposed to 3800 lux od constant white light for 12, 24, and 36 h. During this exposure, the temperature was kept at 28°C and the room was ventilated.

### Cell culture

According to published methods [66, 67], rats were anesthetized with pentobarbital sodium (10mg/kg, Sigma-Aldrich, St. Louis, MO), and olfactory bulbs were removed under a dissecting microscope. Tissue was isolated from the outer nerve and glomerular layers of olfactory bulbs, cut into small pieces, trypsinized (0.1% trypsin for 15 min at 37°C), the process was stopped by incubation in Dulbecco's Modified Eagle's medium/F-12 culture medium (DMEM/F-12, 1:1 mixture, Hyclone) supplemented with 10% fetal bovine serum (FBS, Gibco, Waltham, MA) and a mixture of penicillin and streptomycin. Cells were plated on uncoated 35-mm dishes two times, each for 24 h at 37°C in a humidified atmosphere containing 5% CO_2_. Nonadhesive cells were collected and plated onto 35 dishes coated with poly-D-lysine (PDL, Sigma-Aldrich, St. Louis, MO), and cultured with DMEM/F-12, containing 10% FBS and 1% PS. The culture medium was changed every 3 days. After being cultured for 2 weeks, cells were collected for immunohistochemistry, transplantation, and co-culture experiments. In the following co-culture experiment, 5×10^4^ OECs were seeded per Transwell chamber.

The 661W cells were a gift from Dr Yan Luo [68]. Cells were grown in DMEM (Gibco, Waltham, MA) with 10% FBS at 37°C in a humidified atmosphere containing 5% CO_2_. For the assays, cells were divided into three groups: control, H_2_O_2-_induced, and OEC co-culture groups. In each group, 5×10^4^ cells were seeded per well on a 24-well plate. After being cultured for 24 h, cells in the model group were treated with 0.5 mM H_2_O_2_ for 30 min, and then co-cultured with OECs for an additional 24 h in Transwell chambers.

### Cell transplantation

Rats with retinal damage were used for cell transplantation according to published protocols [38, 40, 69]. One day post 24-h continuous light-induced damage, subretinal injection surgery was performed immediately after an ERG recording. Before surgery, OECs were dissociated into suspension and then labeled with CellTracker CM-DiI(2 μg/ml, Invitrogen, Waltham, MA). Cells were washed twice with PBS and then resuspended in PBS. Under an operating microscope, keratonyxis was performed with a 30-gauge syringe needle to reduce intraocular pressure by increasing the aqueous humour outflow. After penetrating the sclera and choroid, 3 μl of a suspension of labeled OECs (1×10^5^ cells/μl) were slowly injected into the subretinal space of the temporal retina using a 10-μl micro-syringe. Rats in the PBS group were injected with 3 μl PBS.

### ERG test

ERGs were recorded 1 day before and 1 day after light-induced damage and at 2, 4, and 8 weeks after retinal cell transplantation. According to published methods [40, 70], rats were anesthetized with amine (100 mg/kg) and xylazine (12 mg/kg) under dim red light after dark adaptation for 12 h. Rat pupils were then dilated with phenylephrine eye drops (Santen Pharmaceutical Co., Ltd., Japan). Corneal ERG responses were recorded from both eyes simultaneously with gold wire loops. Carboxymethylcellulose sodium eye drops (Allergan, Ireland) were frequently applied to the cornea to prevent dehydration and to allow electrical contact with the recording electrode. Two needle electrodes inserted under the outer layer served as the reference electrodes, whereas the other electrode placed in the tail served as a “ground”. Amplification was at a 0.1–500 Hz band pass without notch filtering, and stimulus presentation and data acquisition were provided using a RETIscan system (Roland, Germany).

### H&E staining

Rats were anesthetized with tribromoethanol (1 mg/kg; Sigma-Aldrich, St. Louis, MO) and perfused with 4 % paraformaldehyde (PFA) as previously described [70, 71]. Eyes were then isolated from their orbits, and the anterior segments were carefully removed. The eye cups were then fixed in 4% PFA for 1h and then removed into 30 % sucrose overnight. Eyes were frozen in OCT compound (Sakura, Torrance, CA) and cut into 10-μm thick segments; slides were stained with H&E and were examined with a microscope.

### Immunocytochemical staining

To identify cultured cells, rabbit anti-S100 antibody (1:500, Sigma-Aldrich, St. Louis, MO) was used as a cell marker to identify OECs and mouse anti-fibronectin antibody (1:500, Abcam, Cambridge, MA) was used as a cell marker to identify ONFs according to our previous protocol [38, 67]. Coverslips were washed three times with PBS, incubated with secondary antibody goat anti-mouse IgG-488 and goat anti-rabbit IgG-568 (Invitrogen, Waltham, MA, USA) for 1h at room temperature and counterstained with DAPI dihydrochloride (Beyotime, China) for 5 min at room temperature. After being washed three times, the coverslips were mounted with Antifade Mounting Medium (Beyotime, China).

ONL thickness was measured in the horizontal position based on previously established protocels [47, 72, 73]. Cell nuclei were counter-stained with DAPI dihydrochloride, and the thicknesses of the ONLs from the optic nerve head were measured at 0.5 mm intervals using the ImageJ program (developed by Wayne Rasband, NIH, Bethesda, MD; available at http://rsb.info.nih.gov/ij/index.html).

For Ki67 staining, the 661W cells were fixed with 4%PFA and permeabilized with 0.5% Triton X-100. After blocking with a 3% bovine serum albumin solution, the cells were incubated with anti-Ki67 antibody (1:500, Abcam, Cambridge, MA) overnight at 4°C. Then the cells were incubated with secondary antibody, goat anti-rabbit IgG-568, for 1h at room temperature and stained with DAPI dihydrochloride. Images were captured with a fluorescence microscope.

### Immunofluorescent staining of ROS in the retina

Retinal intracellular ROS generation was analyzed with a DCFH-DA probe using afrozen sections reactive oxygen species testing kit (Genmed Scientifics Inc., Wilmington, DE). Frozen sections were air dried at room temperature for 15 min and washed with Reagent A. All sections were then incubated with dye containing 1% Reagent B and 99% Reagent C for 20 min at 37°C and then washed with Reagent A. After staining with DAPI dihydrochloride, sections were washed with PBS, cover slipped with antifade mounting medium, and then counted under a fluorescent microscope.

### CCK-8 assay

The cell viability of 661W cells was measured using a cell counting kit-8 assay (CCK-8, Dojindo, Japan). The 661W cells were plated on 24-wells plates(5×10^4^ cells/well), and the OECs were plated in Transwell (5×10^4^ cells). After being cultured for 1 day, 661W cells were treated with 0.5mM H_2_O_2_ for 30 min and then co-cultured with OECs for 24 h by using in a Transwell chamber. Cells were washed with PBS three times, then 300μl fresh DMEM with 30μl CCK-8 solution was added to the 24-well plates. After incubation at 37°C for 1 h, the absorbance was measured at 450nm and 570nm using a microplate reader (Varioskan Flash, Thermo-Fisher, Waltham, MA).

### EdU staining of 661W cells

According to the manufacturer's instructions for the Cell-LightTM EdU staining kit (Ribobio, China), cells were treated with 50μM/L EdU for 2h at 37°C. After fixing with 4% PFA for 20min and treatment with 0.5% Triton X-100 for 10min at room temperature, cells were incubated with Apollo reaction reagent for 30min at 37°Cand counterstained with DAPI dihydrochloride. The images were captured with a fluorescence microscope.

### Apoptosis

Apoptosis of 661W cells was measured using the TUNEL assay, an *in situ* cell death detection kit (Roche, Nutley, NJ) and flow cytometry using an apoptosis detection kit (BD, Franklin Lakes, NJ). Cells were fixed, permeabilized, and incubated with TUNEL reaction mixture at 37°C for 1 h and stained with DAPI dihydrochloride for 5 min at room temperature. Then, the cells were observed and counted under a fluorescent microscope.

For apoptosis assessment, the 661W cells were washed with PBS three times and then harvested and resuspended in binding buffer. Cells were incubated with PI and APC-conjugated Annexin V and then analysed by flow cytometry (BD FACS Calibur, Franklin Lakes, NJ).

### ROS production of 661W cells

To measure ROS production, cells were incubated with DCFH-DA (Sigma-Aldrich, St. Louis, MO) at 37°C for 30 min as described previously. After washing three times with PBS, ROS was measured with fluorescence microscope and flow cytometry.

### Total antioxidant capacity

The total antioxidant capacity of cells was measured by the ferric reducing ability of plasma (FRAP) method using a Total Antioxidant Capacity Assay Kit (Beyotime, China). Briefly, cells were collected, homogenized and incubated with FRAP solution at 37°C for 5 min. The absorbance was measured at 595nm using a microplate reader (Varioskan Flash, Thermo-Fisher, Waltham, MA).

### Real-time PCR

After treatment with H_2_O_2_ and co-culturing with OECs, 661W cells from three independent experiments were collected for subsequent real-time PCR, as previously described [70]. In brief, after total RNAs was isolated with Trizol reagent (Takara, Japan), cDNA was synthesized in 20 μl of reaction mixture from 2 μg of total RNA using a PrimeScript RT reagent Kit (Takara, Japan) according to the manufacturer's instructions. The primers used in the present study indicated in Table 1 and designed using IDT Scitools. Real-time PCR was performed in a Bio-Rad 5-Color System (Bio-Rad Laboratories, Hercules, CA) with a SYBR Premix Ex Tag (Tli RNaseH Plus) kit (Takara, Japan). The results were analyzed using the 2(-ΔΔ C(T)) method.

**Table 1:** Designing real-time PCR primers

Genes(mouse)	Forward primer	Reverse primer
β-Actin	TGAGCTGCGTTTTACACCCT	TTTGGGGGATGTTTGCTCCA
NOX4	GCGTCCTCGGTGGAAACTT	AGTCCCCAGTAGTCTCCTAAG
iNOS	AGTCCCCAGTAGTCTCCTAAG	GAACGTAGACCTTGGGTTTGC
SOD1	GAACGTAGACCTTGGGTTTGC	GAACGTAGACCTTGGGTTTGC
CAT	CCAAGCAATATGCCCCCTGG	GTAATAGTTGGGGGCACCAC

### Western blots

According to our previous protocol [70], 661W cells were lysed in ice-cold RIPA buffer (Beyotime, China) and centrifuged at 12,000xg at 4°Cfor 10 min, and the protein was measured using a BCA protein assay kit (Beyotime, China). In total, 50μg of protein from each group was separated via SDS-PAGE and transferred to PVDF membranes. Blots were blocked with 5% non-fat milk in Tris-buffered saline/Tween for 1 h at room temperature. Then membranes were incubated with primary antibodies (NOX4, Abcam, Cambridge, MA; iNOS, Santa Cruz, Dallas, TX; SOD1, Bioworld, China) overnight at 4°C. After incubation with peroxidase-conjugated immunoglobulin G (1:2,000; Santa Cruz, Dallas, TX) as secondary antibodies, the membranes were scanned using a ChemiDoc MP ImagingSystem (Bio-Rad Laboratories, Hercules, CA).

### Statistical analysis

Data are presented as mean± SEM for at least three independent experiments. Data were evaluated using one-way ANOVA followed by a Tukey's test using SPSS 13.0 software for comparing the means between groups (*p*<0.05 was considered to be statistically significant).

### Compliance with Ethical Standards

All of the animal experimental procedures were subjected to the provisions of the Southwest Hospital Animal Ethics Committee in Chongqing, China.
